# Prediction of Flux and Rejection Coefficients in the Removal of Emerging Pollutants Using a Nanofiltration Membrane

**DOI:** 10.3390/membranes13110868

**Published:** 2023-11-01

**Authors:** Asunción M. Hidalgo, María Gómez, María D. Murcia, Elisa Gómez, Gerardo León, Irene Alfaro

**Affiliations:** 1 Chemical Engineering Department, University of Murcia, Campus Universitario de Espinardo, 30100 Murcia, Spain; maria.gomez@um.es (M.G.); md.murcia@um.es (M.D.M.); egomez@um.es (E.G.); irene.alfaro@um.es (I.A.); 2 Chemical and Environmental Engineering Department, Polytechnic University of Cartagena, 30206 Cartagena, Spain; gerardo.leon@upct.es

**Keywords:** nanofiltration membranes, emerging pollutants, carbamazepine, ketoprofen, bisphenol A, solution-diffusion model, Spiegler-Kedem-Katchalsky model

## Abstract

The removal of three emerging pollutants: carbamazepine, ketoprofen, and bisphenol A, has been studied using the nanofiltration flat sheet membrane NF99HF. The removal efficiencies of the membrane have been evaluated by two system characteristic parameters: permeate flux and rejection coefficient. The influence of two operating variables has been analysed: operating pressure and feed concentration. Before and after the tests with emerging pollutants, the membrane has been characterized by determining its water permeability coefficient and its magnesium chloride rejection coefficient to find out if the removal of emerging pollutants causes membrane fouling. The results show that operating pressure has significant separation effects, obtaining the highest efficiencies at a pressure of 20 bar for pollutant concentrations between 5 and 25 mg/L. Moreover, rejection of ketoprofen was found to be dependent on electrostatic repulsion, while rejection of bisphenol A was significantly affected by adsorption onto the membrane. Finally, the experimental data have been fitted to the solution diffusion model and to the simplified model of Spiegler-Kedem-Katchalsky to predict the behaviour of the nanofiltration membrane in the removal of the tested pollutants. Good agreement between the experimental and predicted carbamazepine and bisphenol A data has been obtained with each model, respectively.

## 1. Introduction

Due to demographic growth in the countries of southern Europe, the demand for and quality of water have been affected, with new chemical substances (such as pharmaceuticals, detergents, personal care products, drugs, and pesticides, among others) increasingly found in surface waters, treated waters, and even in drinking water.

The presence of these compounds in the aquatic environment is relatively recent, as current analytical techniques can detect them at concentrations of ng/L and μg/L. Consequently, their regulation is also relatively new at the European level, starting in 2015, with a focus on registering those that may pose higher risks [1].

The presence of pharmaceuticals and endocrine disruptors has been detected both in the effluent and influent of domestic and industrial wastewater treatment plants. As a result, conventional treatment methods are not sufficient to effectively remove these compounds from the water [2]. This poses a serious issue, particularly concerning the reutilization of regenerated water.

To improve or implement water purification and treatment processes concerning these contaminants, membrane technology proves to be highly effective. Among these technologies, nanofiltration stands out due to its low consumption of reagents and operational costs, showing better cost-effectiveness ratios [3]. Various studies confirm that both Reverse Osmosis (RO) and Nanofiltration (NF) are capable of removing between 82 and 97% of these emerging contaminants (pharmaceuticals, pesticides, and endocrine disruptors) from wastewater [4,5,6]. Furthermore, in potabilization processes, they have been found to reduce the presence of pesticides and Bisphenol A by up to 90% [3], which is advantageous as both are limited in drinking water [7]. An advantage of NF is that it has low energy requirements and a compatible molecular cut size to carry out the removal of emerging contaminants.

Indeed, understanding the phenomena that lead to solute retention in the reject stream and prevent it from passing through the membrane is a topic of interest. Multiple factors can influence the separation/removal mechanisms between the membrane and the solute. It has been described that factors such as the Molecular Weight Cut-Off (MWCO) of the membrane, effective pore radius, contact angle, and isoelectric point, along with the size, shape, and charge of the solute, among others, can significantly affect the separation process [8,9,10,11,12,13,14,15]. These factors play a crucial role in determining the efficiency and selectivity of the membrane in removing specific contaminants from the water. Researching and understanding these aspects are essential for optimising membrane technology and developing better water treatment processes.

On the other hand, mathematical transport models used in separation processes can serve as predictive models, allowing the system to be characterised within a range of process conditions and understanding its behaviour. This, in turn, reduces the number of experimental trials needed to be conducted.

According to the mechanism of solute and solvent transport through membranes, models are classified into different groups [16]. For instance, irreversible thermodynamic models consider that transport occurs through diffusion, driven by the concentration gradient across the membranes, and convection, due to the applied pressure gradient, without relating it to the physicochemical parameters of the membrane. The Spiegler-Kedem-Katchalsky model is a notable example of this category. Another model is the solution-diffusion model, where the physicochemical properties of the membrane are related to the diffusive transport of solute and solvent, considering their independent flows. The solution-diffusion model is a prominent example in this group.

Therefore, the main objective of this work is to study the performance of an NF99HF nanofiltration membrane in removing three emerging contaminants found in surface and drinking waters: two pharmaceuticals, carbamazepine and ketoprofen, and an endocrine disruptor, bisphenol A. The selection of these pollutants was based on their commercial use (carbamazepine and ketoprofen) and their adverse effects on health (bisphenol A). Additionally, two mathematical transport models have been applied to determine which one better predicts the system’s behaviour. Although the removal of these compounds with nanofiltration membranes has been previously tested, this type of membrane (NF99HF) has not been studied yet. The NF99HF membrane has high flux, low fouling, good pH and temperature tolerance, and a molecular weight cutoff (MWCO) appropriate to the contaminants.

## 2. Materials and Methods

### 2.1. Materials

#### 2.1.1. Chemicals

- Magnesium chloride hexahydrate, MgCl_2_ 6H_2_O, 203.30 g/mol, supplied by Panreac (Barcelona, Spain).
- Conductivity standards (147 µS/cm, 1288 mS/cm at 25 °C) were provided by CRISON (Barcelona, Spain).
- Carbamazepine C_15_H_12_N_2_O, 236.27 g/mol, supplied by Sigma-Aldrich (St. Louis, MO, USA).
- Ketoprofen, C_16_H_14_O_3_, 254.28 g/mol (≥98%), supplied by Sigma-Aldrich.
- Bisphenol A, C_15_H_16_O_2_, 228.29 g/mol (≥99%), supplied by Sigma-Aldrich.
- Sodium hydroxide, NaOH, 40.00 g/mol, supplied by Honeywell (Charlotte, NC, USA).
- Absolute ethanol, CH_3_CH_2_OH, 46.07 g/mol, supplied by Panreac.
- Distilled water.

#### 2.1.2. Membrane

The flat nanofiltration membrane NF99HF, used to conduct the experimental series, has been supplied by Alfa Laval, and its specifications are listed in Table 1.

#### 2.1.3. Equipment

- Experimental system

The membrane module used to perform the assays was the Triple System Model F1, manufactured by the commercial company MMS, which has a maximum operating pressure and temperature of 40 bar and 50 °C, respectively.

Through the pump, the feed is driven into the flat membrane module, where the NF99HF nanofiltration membrane has been previously installed. After tangential filtration takes place through the membrane, the feed is divided into two streams: the permeate, collected on a digital balance, and the reject, recirculated back to the feeding tank through a three-way valve. As a result, the unit does not operate continuously, as the permeate stream does not return to the feeding tank.

The pressure required for the filtration process is supplied to the system using nitrogen gas, stored in a cylinder outside the laboratory. Once the main nitrogen valve is opened, the feed pressure is regulated with the three needle valves and the pressure gauge provided by the equipment.

Additionally, there is a laptop computer with an application for controlling the different assays. It displays the pressure and temperature at which the feed enters the module, the transmembrane pressure (TMP), the reject stream pressure, and the module pressure drop (ΔP).

- Analysis equipment

The conductivity meter used to determine the concentration of MgCl_2_ in the feeding solution and in the samples collected from the experimental unit was the CRISON brand, model EC-Meter GLP 31.

The concentrations of carbamazepine, ketoprofen, and bisphenol A in the permeate, reject, and feeding samples were determined using the ultraviolet/visible spectrophotometer from Thermo Scientific, model Evolution 300 (Waltham, MA, USA), at wavelengths of 290 nm, 260 nm, and 300 nm, respectively. The quartz cuvette used has an optical path width of 1 cm and a capacity of 3 mL.

### 2.2. Methods

#### 2.2.1. Operational Procedure

First, the corresponding assay solution is prepared. After introducing a known volume of this solution into the feeding tank, the digital balance and computer are turned on. Once the main nitrogen valve is opened, the experimental unit is powered on, and the pump is started. Then, using the needle valves and pressure gauge, the feed pressure is adjusted to 5 bar. When the pressure stabilizes, as observed on the computer, the stopwatch is started, and permeate samples are taken every 3 min.

After taking two samples at 5 bar pressure, it is increased to 10 bar. In each assay, the feed pressure is varied at 5, 10, 15, and 20 bars, and two permeate samples are collected for each pressure. At 20 bars, samples are taken every 2 min due to the high flow rate.

At the end of the assay, a sample is taken from the feeding tank using the three-way valve. As the tank has been receiving the reject stream, the concentration of the sample will be higher than the initial concentration. Finally, the collected permeate, reject, and feeding solution samples are analysed using either the conductivity metre or the spectrophotometer, depending on the specific procedure. The tests were carried out in duplicate, and each one lasted 20–30 min. All the experimentation was carried out with the same membrane, and when changing the experimental series, after each contaminant, a wash with distilled water was carried out to condition the membrane and eliminate the remains of the previous contaminant.

#### 2.2.2. Analytical Method

The collected samples from the experimental unit are analysed differently depending on the type of assay they originate from.

(a) Assays with distilled water. The permeate mass is obtained using the OHAUS SP2001 balance, and its volume is measured with a graduated cylinder, similarly carried out for each assay permeate.
(b) Assays with MgCl_2_. The concentration of magnesium chloride in the permeate, reject, and feeding samples is determined by measuring their conductivity with the CRISON EC-Meter GLP 31 conductivity meter. Before conducting the measurements, a calibration curve is prepared.

C_MgCl2_ (mg/L) = (Conductivity (mS/cm) − 40.458)/2270.1

(c) Assays with emerging contaminants. The concentrations of carbamazepine, ketoprofen, and bisphenol A in the permeate, reject, and feeding samples are determined by measuring their absorbance in the Evolution 300 spectrophotometer at the wavelength of maximum absorbance (λm) for each compound. In the conducted assays, the feeding solution contained only one contaminant. Individual calibration curves were constructed for each compound before analysing the samples. To perform this, the absorption spectra of each contaminant were determined to find a wavelength, λm, at which the light absorption by the contaminant is noticeable.

The wavelengths of maximum absorption obtained for carbamazepine, ketoprofen, and bisphenol A are 290 nm, 260 nm, and 300 nm, respectively. These λ_m_ values are similar to those used by other authors [19,20]. After determining the λ_m_ for each contaminant, different calibration curves were obtained.

C_carbamazepine_ = A/0.0501   R^2^ = 0.9987

C_ketoprofen_ = A/0.0689   R^2^ = 0.9999

C_bisphenol A_ = A/0.0211   R^2^ = 0.9996

### 2.3. Experimental Series

The different assays conducted can be classified into the following experimental series:

(1) Experimental series for the initial membrane characterization: This series consists of two assays. In the first assay, the feeding tank is filled with distilled water to determine the membrane solvent permeability. In the second assay, a 1 g/L solution of MgCl_2_ is introduced to determine the membrane rejection coefficient towards saline solutions.
(2) Experimental series to determine the membrane behaviour towards the three emerging contaminants: This series comprises 12 assays. For each compound, four assays are conducted, where, in each one, the feeding concentration is kept constant at 5, 10, 15, and 25 ppm while the pressure varies from 5 to 20 bar. The experimental conditions tested—temperature 20 ± 1 °C; range of pressures; and concentrations—were selected taking into account other previous works [21].
(3) Experimental series for the final membrane characterization: This series includes the same assays as the first experimental series, but they are conducted after the experimentation with the emerging contaminants has been completed. This is to account for the possibility that the contaminants may have affected the membrane, altering its permeability and rejection properties.

## 3. Results

### 3.1. Initial Membrane Characterisation

The initial characterisation has been conducted at a macroscopic level, determining the solvent permeability coefficient and the membrane selectivity for the passage of divalent salts.

According to Equation (1):

J_w_ = A_w_ (∆P − ∆П) (1)

When plotting the mass flux of permeate against the hydraulic pressure gradient, a value of 3.035 ×10^−8^ s/m was obtained for the permeability coefficient A_w_. This value is of the same order of magnitude as that obtained by other authors under similar operating conditions [22]. Table 2 presents a comparison of the permeability coefficients of the NF99HF membrane with the solvent.

It can be observed that the differences obtained are a consequence of the pressure and temperature range used in the tests, as well as the type of flow through the membrane, which can vary depending on the experimental equipment used. The highest permeability coefficients are obtained in tests conducted in other studies at pressures higher than 10 bar [21,23].

The membrane selectivity has been determined using saline solutions containing divalent ions. As indicated in the membrane manufacturer specifications, the rejection coefficient can reach up to 99% for divalent ions, while for monovalent ions, it usually does not exceed 70% [24].

Rejection coefficients ranging from 65.8 to 83.8% have been obtained within the applied pressure range of 5 to 20 bar, respectively.

### 3.2. Emerging Pollutants Removal: Carbamazepine, Ketoprofen, and Bisphenol A

Different tests have been conducted by varying the operating pressure between 5 and 20 bar and the contaminant concentration between 5 and 25 ppm to study the influence of these variables on the permeate fluxes and rejection coefficients.

To discuss the way in which emerging contaminants are separated, it is essential to know not only the membrane characteristics (Table 1) but also the physicochemical properties of these contaminants, which are listed in Table 3.

**Figure 1:**
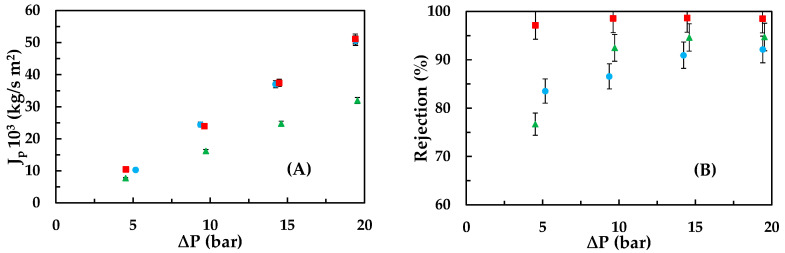
Influence of the hydraulic pressure gradient on the permeate flux (**A**) and rejection coefficient (**B**) for the different emerging pollutants: (●) carbamazepine, (■) ketoprofen, and (▲) bisphenol A at a feed concentration of 15 ppm.

In Figure 1A,B, the permeate fluxes (A) and rejection coefficients (B) are shown for each of the tested emerging pollutants at different operating pressures. As observed in Figure 1A, the permeate flux increases with the operating pressure for all contaminants; these values are consistent with those reported in previous works [32,33]. As the pressure increases, the difference between the permeate flux obtained for carbamazepine and ketoprofen solutions and that obtained for the bisphenol A solution also increases.

Bisphenol A is the pollutant with the highest octanol-water partition coefficient (Table 3). Due to its hydrophobic nature and the prevalence of its neutral form in solution, it tends to adhere to the hydrophobic surface of the NF99HF membrane. This adhesion takes place, causing the permeate fluxes to be lower than those obtained for the other contaminants but not significantly lower due to the small contact angle with the NF99HF membrane, which allows the permeate flux to continue increasing with pressure. This phenomenon has been described by other authors [34,35].

In Figure 1B, it can be observed that the rejection coefficients increase with the operating pressure. In the conducted tests, the membrane surface was negatively charged as the pH was close to 7, which is higher than its isoelectric point [12]. At this pH value, ketoprofen exists mostly in its anionic form, leading to electrostatic repulsion between ketoprofen and the membrane surface, minimizing its adhesion to the membrane. This is because ketoprofen has a high log K_ow_, similar to that of bisphenol A. Consequently, ketoprofen exhibits the highest rejections (very close to 100%) due to the combined effects of electrostatic repulsion and being the contaminant with the highest molecular weight. Hence, it is the only contaminant that does not show significant variation in rejection values within the studied pressure range.

On the other hand, it is observed that carbamazepine has lower rejection coefficients than bisphenol A, despite having a higher molecular weight and being less soluble in water. This could be due to the fact, as established by Van der Bruggen et al. [8], that molecules with a higher dipole moment may have lower rejections than molecules with approximately the same molecular weight but a lower dipole moment. Additionally, bisphenol A has a molecular size represented by its Stokes radius of 0.5 nm, which is larger than carbamazepine’s 0.37 nm and the effective pore radius of the membrane (0.43 nm), favouring its rejection.

**Figure 2:**
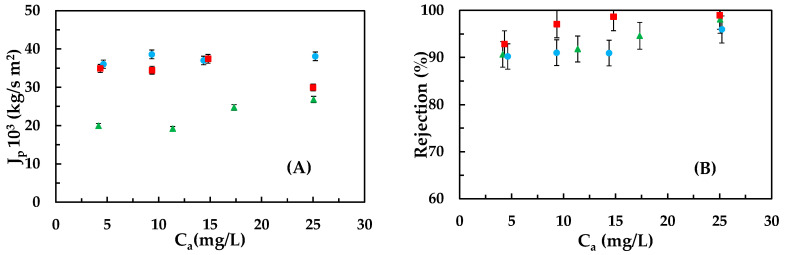
Influence of the feed concentration on the permeate flux (**A**) and rejection coefficient (**B**) for the different emerging pollutants: (●) carbamazepine, (■) ketoprofen, and (▲) bisphenol A at a feed pressure of 15 bar.

In Figure 2A,B, the permeate fluxes (A) and rejection coefficients (B) are shown for each of the tested emerging pollutants at different feed concentrations.

For bisphenol A, the permeate fluxes are lower than those obtained for the other contaminants due to the adhesion phenomenon. The permeate flux does not decrease significantly with concentration because the tests were conducted at low contaminant concentrations, where the osmotic pressure gradient is practically negligible and hardly increases with concentration.

In Figure 2B, it is observed that ketoprofen shows the highest rejections regardless of the feed concentration, followed by bisphenol A and carbamazepine. This indicates that the electrostatic repulsion and adhesion phenomena for ketoprofen and bisphenol A, respectively, do not decrease with the increase in feed concentration from 5 to 25 ppm.

The rejection coefficient for bisphenol A, and consequently, the amount that adheres to and diffuses through the membrane, is generally constant at concentrations above 5 mg/L. Li and Gao [36] observed a decrease in the rejection coefficient, from 90 to 81%, when increasing the concentration of bisphenol A from 0.5 to 3 mg/L. This suggests that a decrease in the rejection coefficient could have been observed if the tests had been conducted in a concentration range lower than 5 ppm, where the amount of bisphenol A adhered to the membrane would increase with the feed concentration until stabilizing, resulting in rejection coefficient values similar to those represented in the figure.

In Table 4, a comparison of the rejection coefficients obtained by other authors, along with those determined in this study under similar operating conditions, is presented.

The experimentally determined rejection coefficient for carbamazepine, at 89.2%, is higher than the one obtained by Kabbani et al. [24]. This difference in rejection could be attributed to the molecular weight cut-off (MWCO) of the NF270 membrane, which is 300 Da, and the test being conducted at 30 °C.

Regarding ketoprofen, there have been few studies on its removal using membrane technology, and the parameters found in the literature often correspond to feed concentrations lower than those studied in this research. Comparing the rejection coefficient determined by Ge et al. [19] with the one obtained under the most similar operating conditions, approximately 85%, it can be observed that the former is higher due to being conducted at a much higher concentration, 4.3 ppm.

The rejection coefficient of 81% for a feed concentration of 3 ppm of bisphenol A reported by Li and Gao [36] is higher than the experimentally determined coefficient. This difference in rejection could be attributed to the membrane used by the authors, which may have a higher contact angle and/or a lower molecular weight cut-off (MWCO) compared to the NF99HF membrane used in this study.

### 3.3. Fouling

With the aim of determining the degree of deterioration, aging, or alteration that the membrane undergoes after conducting the tests with emerging contaminants, new permeability tests with the solvent and selectivity tests with divalent salts have been performed.

The fouling factor F_p_ has been calculated for different operating pressures as the percentage reduction in the mass flow rate of permeate:

(2) $$F_{p}\left(\%\right)=\frac{{\left(Jp\right)}_{0}-{\left(Jp\right)}_{f}}{{\left(Jp\right)}_{0}}\times 100$$

where ${\left(Jp\right)}_{0}$ and ${\left(Jp\right)}_{f}$ are the permeate flows obtained from the initial and final membrane characterisation.

For the divalent salts tests, an additional fouling factor, *F_s_*, has been calculated as the percentage reduction in the solute mass flow:

(3) $$F_{s}\left(\%\right)=\frac{{\left(Qp\cdot Cp\right)}_{0}-{\left(Qp\cdot Cp\right)}_{f}}{{\left(Qp\cdot Cp\right)}_{0}}\times 100$$

where ${\left(Qp\right)}_{0}$ and ${\left(Qp\right)}_{f}$ are the average volumetric permeate flows obtained from the initial and final membrane characterisation and ${\left(Cp\right)}_{0}$ and ${\left(Cp\right)}_{f}$ are the initial and final solute concentrations.

Table 5 shows the fouling factor obtained in the tests with the solvent and the saline solution of magnesium chloride.

Similarly to other studies [38], the highest fouling factor is obtained at a lower operating pressure, which could be due to the pressure being insufficient to overcome the resistance posed by membrane fouling to the passage of the solvent.

By using the saline solutions again, lower permeate fluxes have been obtained compared to the initial ones. This reduction in flux is a consequence of the decrease in membrane permeability to water and the increase in the osmotic pressure gradient across the membrane, both of which result from the narrowing of the pores. As a result, the reduction in the mass flow rate of magnesium chloride that passes through the membrane is a consequence of the decrease in permeate flux and the increase in the rejection coefficient.

### 3.4. Application of Mathematical Models

The experimental values obtained have been fitted to two membrane transport models, discussing whether these models are capable of predicting the characteristic parameters of the system.

#### 3.4.1. Simplified Solution-Diffusion Model

The solution-diffusion models [38] have been used to depict transfer through membranes after experimentally determining the constants of the models. Systems mass balances together with solution-diffusion mass transfer models have been used to simulate the separation process; mainly, this model has been widely used in reverse osmosis membranes, but it has also been applied to nanofiltration membranes [39]. The model equations used in the present work have previously been discussed in other research works [40].

The characteristic equations of the model, 3 and 4, which allow determining the volumetric permeate flow rate (*Q_P_*) as well as the solute concentration (*C_P_*) in it based on the parameters osmotic pressure coefficient (Ψ) and solute permeability of the membrane (B_S_), have been used in other studies [39].

(4) $$C_{p}=\frac{Ca}{1+\frac{A_{w}\cdot ∆P}{B_{s}\cdot C_{w}}-\frac{{\Psi \cdot C}_{a}\cdot A_{w}}{B_{s}\cdot C_{w}}}$$

(5) $$Q_{P}=\frac{A_{W}\cdot S}{C_{W}}(∆P-\Psi \cdot C_{a}+\frac{\Psi \cdot Ca}{1+\frac{A_{w}\cdot ∆P}{B_{s}\cdot C_{w}}-\frac{{\Psi \cdot C}_{a}\cdot A_{w}}{B_{s}\cdot C_{w}}})$$

The solution-diffusion model considers the solute flow (*J_s_*) independent of the solvent flow, obtaining *B_S_* from the slope of Equation (6), while Ψ is determined from Equation (7), derived by other authors [39].

(6) $$J_{S}=B_{S}\cdot (C_{a}-C_{P})$$

(7) $$\left(∆P-\frac{J_{p}}{A_{w}}\right)=\Psi \cdot (C_{a}-C_{P})$$

The values obtained for the solute permeability coefficient and the osmotic pressure coefficient are shown in Table 6.

The determination coefficient obtained for carbamazepine (close to 0) is a result of fitting the experimental data to Equation (7), which yields an almost horizontal line. Overall, low determination coefficients are obtained, probably because this model works better to estimate the removal of inorganic salts in reverse osmosis membranes. However, the significance of applying this model lies in the fact that other authors [21,32,39] obtain high coefficients of determination and accurate estimations of the behaviour of nanofiltration membranes towards organic compounds. For example, this membrane has been used to eliminate methyl paraben, and the determination coefficients are higher [21].

**Figure 3:**
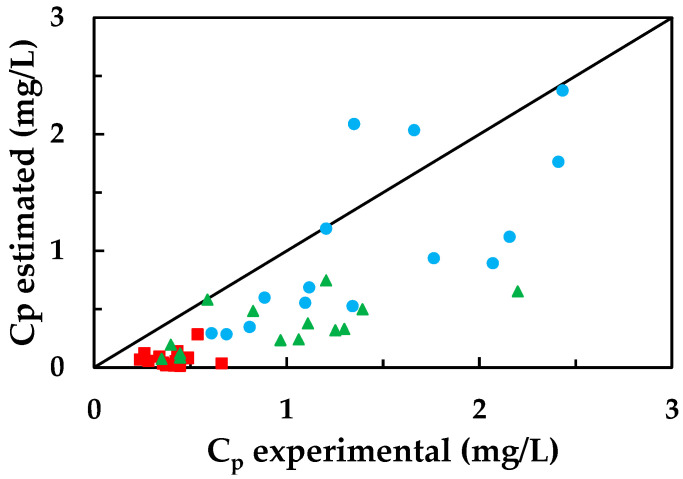
Concentrations calculated with the solution-diffusion model compared to the experimental values for: (●) carbamazepine, (■) ketoprofen, and (▲) bisphenol A.

With the model parameters, the initial permeability of the membrane, and Equations (3) and (4), the permeate flow rate and its concentration are calculated for each of the conducted experiments. Figure 3 depicts the values of the emerging contaminant concentration in the permeate compared to their corresponding experimental values.

The calculated concentrations for ketoprofen and bisphenol A differ from the experimental values. Regarding bisphenol A, this discrepancy may be due to the fact that the model itself does not consider the possibility of convective flow, meaning it does not account for the dragging of bisphenol A adhered to the membrane by the solvent. Similar results are obtained using atrazine solutions and the NF-99 membrane with the same models [39].

Regarding ketoprofen, the lower concentrations compared to the experimental values are a consequence of electrostatic repulsion phenomena that hinder its diffusion through the membrane. Its limited presence in the permeate is likely due to the applied pressure (convective flow).

**Figure 4:**
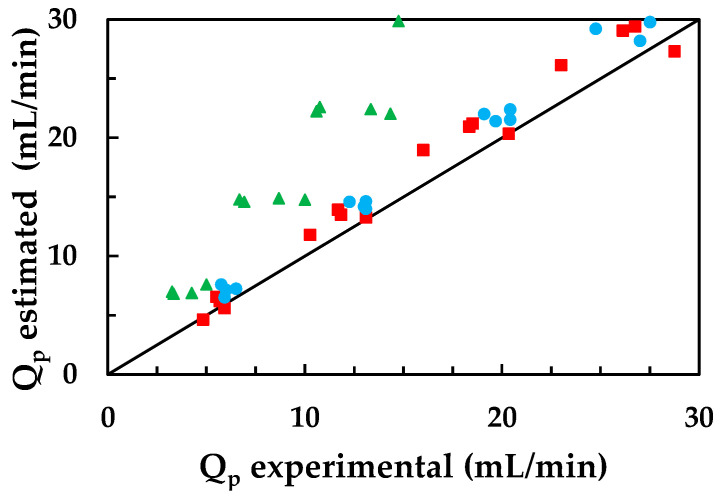
Volumetric permeate flows calculated with the solution-diffusion model were compared to the experimental values for: (●) carbamazepine, (■) ketoprofen, and (▲) bisphenol A.

Similarly, in Figure 4, the values of the volumetric permeate flow rate have been plotted against their corresponding experimental values.

In this case, the calculated permeate flow values for bisphenol A are higher than the experimental values because the model does not consider its adhesion to the membrane, which slightly reduces the permeate flow.

The fact that coherent results were obtained for carbamazepine, with concentrations and flow rates close to the experimental values, indicates that its presence in the permeate depends largely on its diffusion through the membrane. Therefore, the simplified solution-diffusion model is considered valid for estimating the membrane behaviour towards carbamazepine but not capable of predicting its behaviour towards ketoprofen and bisphenol A. Other studies report a good fit between experimental values and values predicted using the Spiegler-Kedem-Katchalsky (SKK) model [21,32].

#### 3.4.2. Simplified Model of Spiegler-Kedem-Katchalsky (SKK)

The Spiegler-Kedem-Katchalsky model explains the transport of chemical species across a membrane through a combination of convective and diffusive fluxes. It was originally designed for reverse osmosis, but studies have demonstrated its potential for nanofiltration under certain conditions [41]. The model equations used in the present work have previously been discussed in other research works [23]. The model establishes a relationship between the volumetric solvent flow rates (Q_v_*)* and the molar solute flow rates (L_S_) that cross the membrane using Equations (8) and (9) [16,32,41,42,43,44].

(8) $$Q_{v}=A_{W}\cdot (∆P-\sigma \cdot ∆\pi )$$

(9) $$L_{S}=P_{S}\cdot \left(C_{m}-C_{p}\right)+\left(1-\sigma \right)\cdot Q_{W}\cdot C_{s}$$

The transport of the solute across the membrane occurs through two mechanisms, as stated in Equation (9): diffusion, resulting from the concentration gradient on both sides of the membrane, and convection, after applying a pressure gradient. Thus, the SKK model does take into account, unlike the simplified solution-diffusion model, the convective transport of the solute.

To obtain the characteristic parameters of the model, the reflection coefficient, σ, and the solute permeability coefficient, P_s_, it is assumed, just like in the solution-diffusion model, that the concentration at the membrane surface is approximately the same as in the feed and also that the solvent flow rate is equal to the permeate flow rate as the latter is highly diluted. Thus, σ and P_s_ are obtained from the slope and the intercept of Equation (10), respectively, which are deduced by other authors [41].

(10) $$\frac{L_{s}}{C_{a}-C_{P}}=P_{s}+\left(1-\sigma \right)\cdot \frac{Q_{V}\cdot C_{s}}{C_{a}-C_{P}}$$

By fitting the experimental values for each emerging contaminant, the model parameters for each of them are determined and presented in Table 7.

As can be seen in Table 7, the lowest permeability coefficient is again obtained for ketoprofen, a compound that shows the highest rejections.

Regarding the reflection coefficient, the closer it is to unity, the more difficulty the solute will have in crossing the membrane. A value equal to zero indicates that the membrane is entirely permeable to the solute [41]. Thus, there is a direct relationship between the rejection coefficient and the reflection coefficient, indicating that the values collected in Table 7 show that bisphenol A is rejected to a greater extent than ketoprofen, contradicting what was obtained experimentally.

With the model parameters known, the rejection is calculated for each of the experiments using Equation (11), obtaining the dimensionless coefficient, F, with Equation (12).

(11) $$R_{calculated}=\frac{\sigma \cdot (1-F)}{1-(\sigma \cdot F)}$$

(12) $$F=e^{-\left(\frac{1-\sigma }{P_{s}}\cdot J_{v}\right)}$$

**Figure 5:**
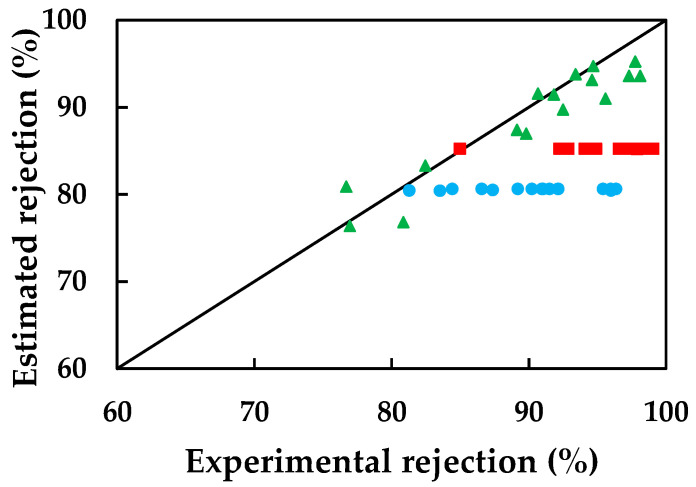
Rejections calculated with the SKK model compared to the experimental values for: (●) carbamazepine, (■) ketoprofen, and (▲) bisphenol A.

In Figure 5, the calculated values of the rejection coefficient have been plotted against the experimental values.

Despite obtaining a reflection coefficient for bisphenol A with a low coefficient of determination and indicating that it has higher rejections than ketoprofen, the SKK model predicts the membrane behavior towards bisphenol A better than the simplified solution-diffusion model. This is because the SKK model takes into account both its diffusive and convective transport through the membrane, as observed by other authors [32,35].

Despite considering convective transport through the membrane, the SKK model may still not accurately predict the characteristic parameters of ketoprofen, likely due to the omission of electrostatic repulsion phenomena between the solute and the membrane surface. This exclusion of electrostatic interactions can lead to an underestimation of theoretical rejection coefficients, resulting in them being lower than the experimental values.

So, the simplified SKK model is considered valid for estimating the membrane behavior towards bisphenol A, as it takes into account both diffusive and convective transport, which is important for this particular solute. However, it may not be able to accurately predict the behavior of the membrane towards carbamazepine and ketoprofen, likely due to the omission of certain specific interactions or phenomena that influence the transport of these solutes.

## 4. Conclusions

After studying the removal of different emerging pollutants with the NF99HF membrane, it has been found that ketoprofen exhibited higher rejection coefficients due to electrostatic repulsion and molecular exclusion phenomena, whereas the rejection coefficient for bisphenol A was higher than that of carbamazepine, attributed to its adhesion to the membrane surface, as described by other authors.

Among the two operational variables studied (concentration and operating pressure), the concentration of the contaminant in the feed stream has the least effect on permeate flux and rejection coefficients. Permeate flux increases with feed pressure, resulting in lower mass flows for bisphenol A as its adhesion hinders solvent passage. As for the rejection coefficients, the values are nearly constant in the case of ketoprofen, and they increase with the operating pressure for carbamazepine and bisphenol A.

In the study of different transport models applied to this work, it has been found that the solution-diffusion model is capable of predicting the characteristic parameters of the system for carbazepine due to its high diffusive transport. On the other hand, the Spiegler-Kedem-Katchalsky model has provided insights into the membrane behaviour towards bisphenol A since it considers both convective and diffusive flow components.

Finally, it is established that the NF99HF membrane can reduce the presence of carbamazepine and ketoprofen in aqueous solutions, achieving the best separation process efficiency at a pressure of 20 bar, regardless of the feed concentration. However, for bisphenol A, obtaining high rejection coefficients but low permeate flux suggests that reducing bisphenol A adhesion to the membrane surface could be a subject of research to achieve higher permeate fluxes and improve rejection coefficients.

## Figures and Tables

**Table 1:** Technical specifications of the NF99HF membrane [17].

Characteristics	Technical Specifications
Manufacturer	Alfa Laval
Name	NF99HF
Type	Thin-layer composite polyester
Composition	Polyamide
MWCO (Molecular Weight Cut-Off) (Da)	≥200 ^b^
pH Range	3–10
Maximum Temperature (°C)	50
Maximum Pressure (bar)	55
MgSO_4_ Rejection (%)	≥98
Isoelectric Point (pH)	4.12–4.42 ^a^
Effective Pore Radius (nm)	0.43 ^a^
Contact Angle (°)	34.5 ± 4.2 ^c^
Effective Surface Area (m^2^)	0.0028

^a^ [18], ^b^ [9], ^c^ [10].

**Table 2:** Permeability coefficients of the NF99HF membrane to water.

A_w_		Temperature (°C)	ΔP (bar)	Flat Sheet Membrane Module Used	Reference
(s/m)	(L/m^2^·h bar)				
6.175 × 10^−8^	22.230	-	10–25	INDEVEN with tangential filtration	[21]
2.961 × 10^−8^	10.661	25	5–30	Alfa Laval Lab M20 with tangential filtration	[22]
4.788 × 10^−8^	17.237	15.8–18.1	10–30	INDEVEN with tangential filtration	[23]
3.035 × 10^−8^	10.927	19.7–20.5	5–20	Triple System Model F1 with tangential filtration	Experimental

**Table 3:** Physico-chemical properties of the studied compounds [25,26,27].

Emerging Pollutant	Carbamazepine	Ketoprofen	Bisphenol A
Molecular structure	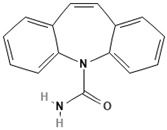	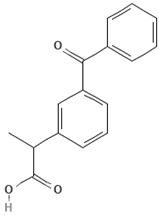	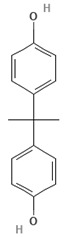
Chemical formula	C_15_H_12_N_2_O	C_16_H_14_O_3_	C_15_H_16_O_2_
Molecular weight (g/mol)	236.7	254.28	228.29
Solubility in water (mg/L)	18	51	120
Dissociation constant	pKa = 13.9	pKa = 3.98	pKa = 9.6
Charge at pH 7	Neutral ^a^	Negative ^b^	Neutral ^a^
log K_OW_	2.45	3.12	3.32
Dipole moment (D)	3.6 ^c,f^	4.37 ^d^	2.13 ^f^
Stokes radius (nm)	0.37 ^c^	-	0.5 ^e^

^a^ [19], ^b^ [20], ^c^ [28], ^d^ [29], ^e^ [30], ^f^ [31].

**Table 4:** Comparison between experimental and bibliographical parameters.

Emerging Pollutant	Membrane	Experimental Conditions	Rejection(%)	J_p_ (kg/m^2^s)	Reference
Carbamazepine	NF270	C_a_ = 10 ppm ∆P = 10 bar	70–80	-	[24]
Carbamazepine	NF270	C_a_ = 200 ppb TMP = 5 bar	80	-	[19]
Ketoprofen	NF270	C_a_ = 200 ppb TMP = 5 bar	93	-	[19]
Bisphenol A	NF90	C_a_ = 50 ppm ∆P = 10 bar	98	-	[37]
Bisphenol A	NF270	C_a_ = 50 ppm ∆P = 10 bar	80	-	[37]
Bisphenol A	Desal5DK	C_a_ = 1 ppb TMP = 20 bar	90–50	-	[35]
Bisphenol A	NF	C_a_ = 0.5–3 ppm ∆P = 4 bar	90–81	-	[36]
Carbamazepine	NF99HF	C_a_ = 9.3 ppm ∆P = 9.7 bar	89.2	24.3	This work
Bisphenol A	NF99HF	C_a_ = 4.2 ppm ∆P = 4.5 bar	77	5.8	This work
Ketoprofen	NF99HF	C_a_ = 4.3 ppm ∆P = 4.5 bar	85	9.9	Thiswork

**Table 5:** Fouling factors obtained in the solvent and saline solution tests at different operating pressures.

ΔP (bar)	5	10	15	20
F_p_ (%)	48.3	40.5	37.2	35.5
F_s_ (%)	64.7	41.7	42.7	49.2

**Table 6:** Solute permeability coefficient and osmotic pressure coefficient values.

	Carbamazepine	Ketoprofen	Bisphenol A
B_s_ (m/s)	3.998 × 10^−6^	3.155 × 10^−7^	7.712 × 10^−7^
R^2^	0.771	0.421	0.774
Ψ (m^2^/s^2^)	8.876 × 10^5^	7.537 × 10^6^	5.657 × 10^6^
R^2^	0.004	0.684	0.362

**Table 7:** Coefficients of solute permeability and reflection.

Coefficients	Carbamazepine	Ketoprofen	Bisphenol A
P_s_ (m/s)	1.368 × 10^−6^	5.647 × 10^−8^	1.472 × 10^−6^
σ	0.841	0.930	0.987
R^2^	0.832	0.617	0.418

## Data Availability

The data presented in this study are available on request from the corresponding author. The data are not publicly available due to they are part of a much larger study that is still underway.
